# Role of adenosine stress perfusion CMR in guiding clinical decision making in pediatric and congenital cardiology: a single pediatric center experience

**DOI:** 10.1186/1532-429X-16-S1-P128

**Published:** 2014-01-16

**Authors:** Hopewell Ntsinjana, Oliver Tann, Marina Hughes, Silvia Schievano, Vivek Muthurangu, Andrew Taylor

**Affiliations:** 1 Institute of Cardiovascular Science, University College London and Great Ormond Street Hospital for Children NHS Foundation Trust, London, UK

## Background

Compared to adults, coronary artery disease abnormalities, significant enough to warrant viability assessment and adenosine stress perfusion cardiac magnetic resonance (CMR) imaging, are rare in pediatric patients. As a result, the role of adenosine stress perfusion CMR as a routine clinical imaging tool in these patients has not been clearly defined. We aim to evaluate the impact clinically-indicated adenosine stress perfusion CMR on decision-making and follow-up strategy.

## Methods

Medical records and CMR images of all adenosine stress perfusion CMR studies performed on pediatric patients between August 2009 and May 2013 at a single institution were retrospectively reviewed. All examinations were performed on a 1.5T Siemens Avanto MRI scanner. Patients </=10 years old were examined under general anesthesia. Adenosine stress perfusion CMR protocol included adenosine at 140 μg/kg/min for a target heat rate increase of 20% or infusion duration of up to 5 minutes and gadolinium of 0.1 mmol/kg. Institutional review board approved this study and all patients signed informed consent.

## Results

Twenty-seven patients were enrolled (median age 14 years, range 1.4-18.2 years). Twenty-six patients completed the protocol with one study terminated due to extreme chest discomfort and nausea. In all completed studies, a heart rate response of >20% was achieved. List of diagnoses and reasons for referral are reported in Table 1. 31% (8/26) of the patients had areas of inducible ischemia. Table 2 displays cardiac diagnoses, anatomical lesion on the coronary artery as proven by CMR or subsequent conventional x-ray angiography and clinical decision made after the results of the perfusion study for these patients. Two of the eight patients with areas of inducible ischemia underwent successful revascularisation, and repeat perfusion studies performed after the intervention showed no evidence of inducible ischemia.

**Table 1:** Cardiac diagnosis and reason for referral.

Cardiac diagnosis	N	Reason for Referral (n)
ALCAPA post Coronary re-implantation	6	Chest pain (3) Routine (3)

Kawasaki disease	6	Abnormal echocardiogram (6)

Transposition of Great arteries post arterial switch	4	Chest pain (3) Abnormal EKG on exercise (1)

Dilated Cardiomyopathy	3	Abnormal EKG (2) Abnormal echocardiogram (1)

Homozygous familial hypercholesterolemia	2	Clinician's request (2)

Atretic left anterior descending coronary artery	1	Sudden collapse during exercise

Bicuspid aortic valve	1	Dyspnea on exercise

Aortic stenosis post Ross procedure	1	Chest pain

DORV post repair	1	Chest pain

Hodgkin's lymphoma post chemo and radio therapy	1	Chest pain

Takayasu's arteritis	1	Cardiac arrest

ALCAPA (Anomalous left coronary artery from pulmonary artery), EKG (Electrocardiogram), DORV (Double outlet right ventricle)

**Table 2:** Clinical Outcome of Positive Stress Test.

Patient	Age (years)	Cardiac diagnosis	Anatomical coronary lesion	Clinical decision
1	9 years	ALCAPA post repair	Focal stenosis of re-implanted left main coronary artery	Plan for surgical re-vascularization

2	14 years	TGA post ASO	Mild ostial stenosis of left main	Limit strenuous exercise

3	10 years	Kawasaki	Aneurysmal left main stem	Medical therapy

4	10 years	Kawasaki	Left main stem stenosis	Endovascular coronary stent implantation

5	15 years	Kawasaki	Aneurysmal LAD	Medical therapy

6	18 years	ALCAPA post repair	Not detected	Plan for coronary angiography

7	12 years	ALCAPA post repair	Focal stenosis of re-implanted left main coronary artery	After x-ray angiography increased follow-up surveillance

8	14 years	Takayasu's arteritis previous LIMA graft	Obstructed LIMA graft	Exercise testing

ALCAPA (Anomalous left coronary artery from pulmonary artery), TGA (Transposition of great arteries), ASO (Arterial switch operation), LAD (Left anterior descending artery), LIMA (Left internal mammary artery).

## Conclusions

This retrospective study demonstrates that adenosine stress perfusion CMR performed in pediatric patients can positively direct clinical decision-making, Lack of ionising radiation makes this an attractive investigation with which to monitor response to revascularisation.

## Funding

The authors would like to acknowledge the support received from Commonwealth Scholarships, UK National Institute of Health Research (NIHR), Royal Academy of Engineering and EPSRC. This report is independent research by the National Institute for Health Research Biomedical Research Centre Funding Scheme. The views expressed in this publication are those of the authors and not necessarily those of the NHS, the National Institute for Health Research or the Department of Health.

